# Editorial: Personalized Nutrition

**DOI:** 10.3389/fnut.2021.669307

**Published:** 2021-03-25

**Authors:** Ellen E. Blaak, Helen M. Roche, Lydia A. Afman

**Affiliations:** ^1^Department of Human Biology, School for Nutrition and Translational Research in Metabolism, Maastricht University, Maastricht, Netherlands; ^2^School of Public Health, Physiotherapy and Sport Science, UCD Institute of Food and Health, UCD Conway Institute, University College Dublin, Dublin, Ireland; ^3^Nutrition, Metabolism and Genomics Group, Division of Human Nutrition and Health, Wageningen University and Research, Wageningen, Netherlands

**Keywords:** personalized nutrition, precision-based nutrition, microbiome, web-based intervention, childhood, plant-based diets

Worldwide, the prevalence of obesity has grown dramatically since the 1980s (http://www.who.int/news-room/fact-sheets/detail/obesity-and-overweight). Driven by easy access to energy-dense foods and a sedentary lifestyle, obesity has become a major global health and socio-economic problem in the twenty-first century (1), leading to insulin resistance (IR), a pro-inflammatory and hypoxic milieu, and type 2 diabetes (T2D) (2). More recently there has also been concern that the prevalence of obesity-related cancers will rise. While dietary guidelines are evidence-based and valuable for the general population to maintain health, it has become increasingly clear that it may be important to personalize dietary advice to obtain more effective prevention and treatment of chronic metabolic diseases. Indeed, lifestyle intervention, focused on personalization of general guidelines for a healthy diet and physical activity at an individual level, may reduce diabetes and metabolic syndrome risk by more than 50% in different settings worldwide (2–4). Nevertheless, within these tailored interventions, 30% of the participants do not respond and/or adhere to the intervention. More insight is required on factors determining adherence to a healthy diet, as well as the biological mechanisms underpinning divergent responses to dietary interventions, with respect to metabolic outcomes and subsequent potential pathologies, in order to optimize the effectiveness of intervention.

In the Research Topic *Personalized Nutrition*, important dietary strategies are addressed that focus on the delivery of personalized advice, web-based vs. face-to-face (Al-Alwadhi et al.), precision-based strategies, methodological considerations based on diet-microbiome interactions (Johnson et al.; Nestel et al.), insight on mechanisms underlying impaired glucose metabolism in childhood and puberty (Cominetti et al.), as well as a study design of a Randomized Controlled Trial focused on the impact of plant-based vs. animal-based diets (Dawczynski).

In the review of Al-Awadhi et al., evidence for the effectiveness of web-based and face-to-face dietary interventions on dietary change is assessed to give more insight into which method may be most effective at delivering personalized nutrition. In total, 19 peer-reviewed randomized controlled trials were identified for inclusion in the review. Findings from personalized web-based nutrition interventions showed that they may be successful at inducing short-term dietary change. Nevertheless, there appears to be insufficient evidence that personalized web-based interventions are as effective as personalized face-to-face interventions, indicating the need for further controlled comparative studies and cost-benefit analyses.

In addition to focusing on the delivery of personalized advice, more information is required on whether precision-based strategies focused on a metabolic or microbial phenotype may be effective at improving the success of nutritional intervention with respect to metabolic health (5).

Microbial responses to foods are very personalized. A landmark study within this field by Zeevi et al. (6) showed that, despite high interpersonal variability in post-meal glucose profiles, personalized diets created with the help of a machine learning algorithm, including data reflecting dietary habits, physical activity, and gut microbiota, may be used to successfully resolve elevated postprandial glucose responses. Additionally, a 2020 study [PREDICT, (7)] again showed that there is large variation in how individuals respond to different meals in a large, well-characterized cohort. Subsequently, the influence of person-specific factors, such as gut microbiome and genetics, on their response was estimated, as was the influence of meal macronutrient composition. Nestel et al. studied whether postprandial glucose response 60 min after a standardized breakfast (PPGR) was associated with gut microbial α-diversity (primary outcome) and explored whether postprandial responses of glucose and insulin were associated with specific microbiome taxa, colonic fermentation markers, and abiotic factors in 31 healthy individuals. They observed that the gut microbiome, measures of colonic fermentation, and abiotic factors (fecal characteristics and intestinal transit time) were not shown to be significantly associated with variability in postprandial responses. This may be related to the fact that contributions may be subtle. Indeed, the PREDICT study showed that the gut microbiome explained just 6% of the variability in glycemic responses (7). However, the sample size is relatively small in the Nestel study. Nevertheless, interesting associations were observed between intestinal transit time and fasting glucose, as well as between fasting breath hydrogen and fasting insulin, which may require further study.

Studying diet-microbiome-host metabolism is complex due to high interindividual variation in both dietary intake and microbial composition, and the difficulty of controlling diet and microbiome covariation in observational and interventional studies. In the excellent review by Johnson et al., variables that should be considered and controlled for in study designs are discussed. The review explores several other factors that need to be considered in future studies, including the requirement of multiple consecutive microbiome samples per study time point or phase and multiple days of dietary history prior to each microbiome sample whenever feasible. Additionally, the difficulty in quantifying dietary intake and analyzing microbial composition is discussed. Although the effects of diet on the microbiome may be observed within a day, subsequent impacts on host metabolism may take much longer to occur. The authors also recognize and review that diet-microbiome interactions may be personalized with consequences for initial stratification and/or sample size.

Personalized or precision-based strategies may also be relevant for different life stages since consumer needs, as well as health profiles, may vary across the lifespan. The obesity pandemic is also of great concern in children, with 124 million children worldwide currently obese (https://www.who.int/end-childhood-obesity/en/). Importantly, Cominetti et al. addressed how temporal glycemic variations during childhood relate to physiological changes in central energy metabolism and substrate oxidation in the EarlyBird study. The EarlyBird study is a non-interventional, prospective cohort study that recruited 307 healthy UK children at age 5, and followed them annually throughout childhood for 12 years with longitudinal data on blood metabolite profile, anthropometry, and respiratory exchange ratio. They showed that fasting glycemia increases steadily during childhood, which is accompanied by increased insulin concentrations, and was positively associated with LDL and VLDL blood lipid signature, as well as greater fasting respiratory exchange ratio, reflecting a shift toward carbohydrate oxidation. Metabolome data show increased flux through glycolytic pathways, and complex changes in amino acids. These findings raise an important question: at what point do physiological changes, such as increasing fasting glycemia, begin to have pathophysiological consequences on cardiometabolic health? Greater understanding of the relation to the underlying mechanisms and pathogenic consequences may provide insights in to the relation with optimal intervention “windows” or time points.

Currently, consumers are driving demand for more plant-based diets and lifestyles in Europe. However, not much is known on the long-term effects on cardiometabolic health of specific lifestyles like flexitarians, vegetarians, and vegans. The biological impact of plant-based dietary interventions is very difficult to implement, wherein isocaloric dietary control groups allow true definition of relative efficacy, independent of energy and macronutrient composition. In the article by Dawczynski, a study design is presented of the NuEva study. This study aims to reveal the impact of chosen nutritional habits (Western diet, flexitarians, vegetarians, vegans) on health status and disease risk with respect to physiological benefits or possible pathophysiological consequences, resulting from long-term implementation of the studied diets. It is a 12-month parallel-designed trial (with 12 months follow-up) with at least 55 participants for each diet (vegetarian, vegan, flexitarian [rare meat/sausage consumption, once or twice per week]), and participants who consume a traditional Western diet as the control group. Major questions that will be answered are: Is it possible to ensure an adequate intake of all essential nutrients; are land-based n-3 PUFA from linseed oil a suitable source to ensure an adequate status of n-3 LC-PUFA; and what impact has each diet had on health and disease and in particular, cardiovascular risk? This study may provide important information on more personalized guidance of groups with specific dietary habits.

In conclusion, it is increasingly evident that one size does not fit all. Thus, personalized or precision-based dietary intervention strategies provide the opportunity to increase the effectiveness of dietary prevention and treatment of chronic metabolic diseases. The present Research Topic addresses different interesting aspects related to personalized delivery of dietary guidance, metabolic profiling of specific age groups, as well as precision-based strategies and methodological considerations based on diet-microbe-host interactions. Overall, we still need more research on the most optimal implementation of dietary advice as well as to relating (epi)genetic, microbial, and metabolic phenotypes to intervention outcomes to define more optimal diets for individuals with or predisposed to chronic metabolic diseases. In this, the different etiologies toward T2D, cardiometabolic diseases, and possibly obesity-related cancers have to be considered. More prospective trials are required to provide the evidence for the implementation of these approaches.

## Author Contributions

EB wrote the editorial. All authors revised the manuscript.

## Conflict of Interest

The authors declare that the research was conducted in the absence of any commercial or financial relationships that could be construed as a potential conflict of interest.
